# Does Video-Assisted Thoracoscopic Surgery with Bullectomy and Partial Pleurectomy for Primary Spontaneous Pneumothorax Impair Health-Related Quality of Life and Pulmonary Function?

**DOI:** 10.3390/healthcare9111463

**Published:** 2021-10-29

**Authors:** Stephen Fung, Hany Ashmawy, Anja Schauer, Martin Eichler, Sami Safi, Levent Dizdar, Alexander Rehders, Wolfram Trudo Knoefel, Georg Fluegen

**Affiliations:** 1 Department of Surgery, University Hospital Duesseldorf, Heinrich-Heine-University Duesseldorf, Moorenstrasse 5, 40225 Duesseldorf, Germany; stephen.fung@med.uni-duesseldorf.de (S.F.); Hany.Ashmawy@med.uni-duesseldorf.de (H.A.); AnjaMaria.Schauer@med.uni-duesseldorf.de (A.S.); Sami-Alexander.Safi@med.uni-duesseldorf.de (S.S.); Levent.Dizdar@med.uni-duesseldorf.de (L.D.); Rehders@med.uni-duesseldorf.de (A.R.); Georg.Fluegen@med.uni-duesseldorf.de (G.F.); 2 National Center for Tumor Diseases (NCT/UCC), University Hospital Carl Gustav Carus, TU Dresden, 01307 Dresden, Germany; Martin.Eichler@uniklinikum-dresden.de

**Keywords:** VATS, quality of life, pulmonary function

## Abstract

Background: Video-assisted thoracoscopic surgery (VATS) with partial pleurectomy is an established treatment for primary spontaneous pneumothorax (PSP). However, postoperative pulmonary function and health-related quality of life (HR-QoL) after VATS–bullectomy with partial pleurectomy (VBPP) have not been elucidated. Methods: Eligible patients were assessed for HR-QoL using the Short-Form 36 (SF-36) health survey. Pulmonary function (PF) was evaluated by spirometry. We compared the results of the VBPP cohort with the German national norms, and with a similar cohort of patients successfully treated by chest tube (CT) only. Results: A total of 25 VBPP patients completed the SF-36 health survey, of whom 15 presented for PF assessment. Between the VBPP and CT groups, the mean forced vital capacity (FVC), forced expiratory volume in one second (FEV1), and FEV1/FVC ratio were not statistically significantly different. However, in both groups, FVC, FEV1, and FEV1/FVC were above the lower limit of normal (LLN), suggesting no restrictive or obstructive patterns. Compared with the sex- and age-matched normal German population, patients who underwent VBPP displayed a similar physical component summary score and a significantly decreased mental component summary score. Interestingly, comparison of the SF-36 domains between the VBPP and CT groups showed no statistical difference. Conclusion: VBPP is a suitable surgical treatment for PSP, with no apparent adverse impacts on pulmonary or physical function. However, psychological distress and measures to counteract its impact should be considered.

## 1. Introduction

According to the German S3 guidelines, primary spontaneous pneumothorax (PSP) describes the presence of air within the pleural space of patients under 45 years of age, without preceding trauma or underlying pulmonary disease [1]. The incidence of PSP has been reported at 1–9.8 and 7–24 cases per 100,000 individuals per year in females and males, respectively [2,3]. Due to the low recurrence and morbidity rates, current guidelines recommend video-assisted thoracic surgery (VATS) for surgical treatment of PSP in cases with ipsilateral recurrence or persistent air leakage after pleural drainage [1,4,5].

VATS is a safe and well-established technique for surgical management of PSP [6]. In previous studies, VATS–bullectomy alone for PSP was demonstrated to have recurrence rates of up to 20% [7,8]. When combined with pleurectomy, the short- and long-term recurrence rates were reduced to 1–6% [6,9,10,11]. To date, studies that evaluate preoperative lung function of patients at first presentation of PSP are lacking. This is most likely due to the acute symptoms at presentation, which might be life-threatening and may require emergency treatment. Additionally, reports that elucidate the impact of VATS, particularly in combination with bullectomy and pleurectomy on pulmonary function after treatment are lacking. It is thus still unknown whether wedge resection of blebs or pleurectomy to ensure tight adhesion of the affected lung to the thoracic wall cause restrictive pulmonary function impairment. Due to the acute symptoms at presentation, pretreatment data were not collected in this study. Thus, we assessed the pulmonary function of our patients following VBPP and CT treatment.

Moreover, any treatment that impairs the social, mental, and physical health of a patient may severely affect their quality of life (QoL). Various surveys have been developed to evaluate the health status and quality of life of patients affected by administered treatments. The SF-36 health survey is a well-established and reliable survey suitable for use in clinical practice and for research [12,13]; it has been widely used in patients of various ages, diagnoses, and nationalities to compare the effects of diseases and the benefits of various treatments [14,15,16]. Regarding surgical treatment of PSP, changes in quality of life after VATS have rarely been reported [17]. Furthermore, the impact of additional bullectomy and partial pleurectomy on the health-related quality of life (HR-QoL) remains unknown.

Therefore, the aim of this study was to evaluate the HR-QoL and postoperative pulmonary function of PSP patients following VBPP in our institution, and to compare our results with a similar group of patients successfully treated with chest tube drainage only. Additionally, we compared the results of our VBPP cohort with the sex- and age-matched normative data from the general German population.

## 2. Materials and Methods

This was a prospective non-randomized single-centre study. The local Institutional Review Board of the Heinrich Heine University Clinic Dusseldorf approved this study (ref Nr: 2020-1271). Between January 2017 and December 2019, 34 patients underwent VATS–bullectomy with partial pleurectomy due to PSP in our hospital. All patients presented in our emergency room with severe symptoms, and were scheduled for emergency or expedited operation. The patients were contacted one year after surgery and assessed for HR-QoL and pulmonary function at our outpatient clinic. Of these patients, 25 patients with a mean age of 26.1 years (range 17–42) completed the survey and were included in this study; 15 of those 25 patients presented in our outpatient clinic, and were assessed for pulmonary function. For comparability, and to explore the impact of VBPP on pulmonary function and HR-QoL, a control group of 25 eligible patients with mean age of 27 years (range 19–40), who underwent successful chest tube (CT) treatment between January 2018 and April 2020, were included in this study. For pulmonary function, data on 15 patients were evaluated. Likewise, these patients were contacted one year after CT treatment and assessed for HR-QoL and pulmonary function at our outpatient clinic

### 2.1. Surgical Procedure: VATS–Bullectomy with Partial Pleurectomy

All patients in this study underwent the same surgical procedure. In all cases, partial pleurectomy and bullectomy of one or more lung segments was performed. VATS was carried out under general anaesthesia with a double-lumen tube intubation and single lung ventilation. After lateral positioning of the patient, thoracoscopy was performed in the conventional two- or three-port approach. Bullectomy was carried out via wedge resection using an endoscopic stapling device (Autosuture GIA Universal; COVIDIEN^TM^, Mansfield, MA, USA). Partial pleurectomy was performed beginning from the apex of the pleural cavity up to the 7th or 8th intercostal space. An underwater air-leak test was carried out to verify the lack of residual air leaks. One 24 French (Fr) chest tube (COVIDIEN^TM^, Mansfield, MA, USA) placed at the apex of the thoracic cavity was inserted through the trocar incisions at the 5th intercostal space. The chest tube was connected to a digital chest drainage system (Thopaz+, Medela AG, Baar, Switzerland) with a suction equivalent to −20 cm H_2_O.

### 2.2. Assessment of Pulmonary Function

Pulmonary function was assessed via spirometry (EasyOne™ Spirometer, ndd Medical Technologies, 2 Dundee Park, Andover, MA 01810, USA) performed by an experienced PA. The results were measured and interpreted as recommended by the ATS/ERS Task Force [18] and Johnson et al. [19], respectively. The lower limit of normal (LLN) was defined as below the fifth percentile of spirometry data obtained from the Third National Health and Nutrition Examination Survey of age- and physiology-matched controls, calculated using http://hankconsulting.com/RefCal.html (accessed on 27 August 2021). (see Johnson et al. [19]). FVC and FEV1 were expressed in litres, and the FVC/FEV1 ratio as a percentage. To determine any lung function impairment, the predicted FVC and FEV1 values, as well as the FEV1/FVC ratio, were compared with the LLN values. FVC and FEV1/FVC ratio ≥ LLN suggested normal lung function [19].

### 2.3. SF-36 Health Survey

The SF-36 health survey is a self-administrated questionnaire consisting of 8 domains with a set of 36 items that evaluate mental and physical health together. These domains are: physical functioning (PF), measuring limitations in physical activities related to health problems; role physical (RP), assessing role limitations due to physical problems; bodily pain (BP); general health perception (GH); vitality (VT), which represents energy and fatigue; social functioning (SF), reporting on limitations in social activities because of physical and emotional problems; role emotion (RE), measuring limitations in usual role activities due to emotional problems; and, lastly, mental health (MH), representing psychological distress and wellbeing. The sum of the questions in each domain is transformed into a 0–100 scale, with 0 representing the worst and 100 the best health status. The eight domains are condensed into two summary scores: the physical component summary (PCS), and the mental component summary (MCS). The PCS includes the PF, RP, BP, and GH domains. The MCS includes the VT, SF, RE, and MH domains. Results from our questionnaire were re-evaluated as described in the SF-36 Health Survey Manual [20].

### 2.4. Statistical Analysis

Statistical analysis was performed using Microsoft Excel and SPSS 25.0 (IBM Corp, released 2017, IBM SPSS Statistics for Windows, Version 25.0., IBM, Armonk, New York, NY, USA). Clinical data were presented as number, percentage, mean, and standard deviation. The means of variables were compared using Student’s *t*-test. Statistical significance was considered at *p* < 0.05. The scores in each domain were calculated as recommended in the SF-36 Health Survey Manual [20]. The quality of life of patients in each domain was presented as mean and standard deviation. A positive or negative difference between the domains of our data and the normative data from the general German population indicated an increased or decreased HR-QoL, respectively. Normative data from the general German population (1998) were derived from the work of Morfeld et al. (2011) [20]. To obtain age- and sex-matched normative values, we weighted the data in Tables E13, E14, and E19–E22 of the manual [20] according to the number of study participants from the respective age and sex groups.

## 3. Results

Patients’ clinical data (Table 1)—such as age, sex, weight, height, body mass index (BMI), history of pneumothorax, postoperative complications, and smoking history—were retrieved from the medical records. Age, weight, height, and BMI were re-evaluated at presentation in our outpatient clinic

### 3.1. Pulmonary Function

A summary of the pulmonary function data is presented in Table 2. Comparison of the calculated values of FVC, FEV1, and FEV1/FVC ratio between the VBPP and CT groups displayed no statistically significant differences. Interestingly, the values of FVC, FEV1, and FEV1/FVC in both groups were higher than the corresponding estimated LLN values (Table 2), suggesting no restrictive or obstructive pulmonary function impairment with either treatment modality.

### 3.2. SF-36 Scores of Patients Who Underwent VBPP

Seven of the eight domains of the SF-36 health survey of our VBPP cohort were lower compared to those of the general German population. The affected domains included PF, GH, VT, SF, RF, RE, and MH. For these domains, the following differences were evaluated: PF = −3.8, GH = −3.2, VT = −7.0, SF = −0.5, RP = −3.8, RE = −3.2, and MH = −7.0 (Table 3). However, only the domains vitality (VT) (*p* = 0.0460) and mental health (MH) (*p* = 0.0271) were statistically significant. Interestingly, the physical component summary (PCS) scores were similar, whereas the mental component summary (MCS) score was significantly lower (*p* = 0.0049) in our patient cohort compared to the general German population (Table 3). Additionally, there were no significant changes in quality of life between the VBPP and CT groups.

## 4. Discussion

VATS–bullectomy with partial pleurectomy (VBPP) for blebs is a well-established and increasingly used surgical treatment for PSP. Recent studies have reported superior performance of VATS–bullectomy with partial pleurectomy (VBPP), with low rates of recurrence compared to VATS–bullectomy alone [6,9,10,11]. However, the impact of VBPP on the quality of life and pulmonary function of PSP patients is hitherto unknown. In the literature, various studies have reported the impact of VATS and lung resection on HR-QoL and pulmonary function after treatment of malignant diseases such as lung cancer. Avery et al. [21] reported a considerable detrimental impact on patients’ HR-QoL following VATS with lung resection for lung cancer, which was not fully resolved 12 months post-surgery. Veronesi et al. [22] reported a better quality of life for the first year after VATS for lung cancer. Moreover, in two recent studies [23,24], VATS with sublobar resection for lung cancer was associated with preserved lung function. To date, only one study by Balduyck et al. [17] has evaluated the QoL following VATS for a benign disease; in this non-randomized prospective study of 20 patients, VATS and anterolateral thoracotomy for wedge resection and apical pleurectomy achieved comparable results in terms of QoL [17].

In our study, 25 patients who underwent VATS–bullectomy with partial pleurectomy (VBPP) were included. The aim of our study was to verify the impact of VBPP on pulmonary function and HR-QoL one year after surgery. We compared our results with patients successfully treated by chest tube only in our institution, and with normative data from the general German population.

Of the VBPP cohort, we were able to evaluate the lung function of 15 patients at our outpatient clinic (10 patients declined to participate). We found no significant differences in FVC or FEV1, nor in the FEV1/FVC ratio, between the VBPP and CT groups. Interestingly, in both groups, the estimated FVC, FEV1, and FEV1/FVC ratio were higher than the matched LLN values (Table 2), indicating normal pulmonary function after VBPP. This suggests that partial pleurectomy, which causes adhesion of the visceral pleura to the inner surface of the thoracic cavity in order to prevent recurrent pneumothorax, has no restrictive effect on lung function. Furthermore, these results indicate no obstructive pattern (both FVC and FEV1/FVC > LLN). Together, these results demonstrate that VBPP does not adversely affect the pulmonary function of PSP patients, while it was previously shown to be highly effective at reducing the risk of recurrence of PSP.

We used the well-established SF-36 health survey to evaluate the HR-QoL [12,13]. Of 34 patients, 25 completed the SF-36 questionnaire (9 patients were excluded due to an incomplete questionnaire). We compared our results with a control group of patients successfully treated by chest tube only at our institution, and with the normative data from the general German population, as published by Morfeld et al. [20]. For our VBPP cohort, the physical component summary (PCS) scores were similar to the scores of the general German population, indicating that VBPP does not impair physical health. However, the mental component summary (MCS) scores of our patients were significantly lower (score −4.5, *p* = 0.0049) than those of the general German population, indicating high psychological distress for VBPP patients. This may be due to increased stress and post-traumatic anxiety of a possible relapse after surgery. Moreover, we compared the results of the SF-36 survey of the VBPP and CT groups. Interestingly, we found no significant statistical differences between the eight domains and summary scores of the SF-36 survey.

This study is limited by the lack of baseline data (both SF-36 and pulmonary function) and the lack of a surgical control group (e.g., patients treated by VATS without pleurectomy and/or bullectomy). We assessed HR-QoL and pulmonary function in our cohorts one year after surgery/chest tube treatment, and not at different intervals (e.g., 3, 6, and 12 months); this limits our results to a specific period. Additionally, due to the severe symptoms of the PSP patients who presented in our emergency room, a preoperative SF-36 health survey would not have been feasible.

Nevertheless, this study is the first to report on the impact of VBPP on the HR-QoL and pulmonary function of PSP patients after surgery and chest tube treatment. More prospective studies with a larger number of patients are needed in order to evaluate similar outcomes.

## 5. Conclusions

Our data demonstrate for the first time that VATS–bullectomy with partial pleurectomy (VBPP) for primary spontaneous pneumothorax (PSP) has no adverse impact on pulmonary function, and is associated with stable physical health. However, psychological distress and measures to counteract its impact should be considered after surgical treatment.

## Figures and Tables

**Table 1:** Clinical characteristics of the VBPP and CT groups.

	VBPP Group (*n* = 25)	CT Group (*n* = 25)
Gender		
Male	20 (80)	22 (88)
Female	5 (20)	3 (12)
Age mean (range) years	26.1 (18–42)	27 (19–40)
Height (m)	1.8	1.8
Weight (Kg)	64.9	65.7
BMI (kg/m^2^)	19.9	20.1
Smoking history		
Smokers	3 (12)	5 (20)
Non-smokers	22 (88)	20 (80)
History of pneumothorax		
First episode	19 (76)	21 (84)
Recurrence	6 (24)	4 (16)
Complications		
Hemothorax	1 (4)	0 (0)
Prolonged air leak	5 (20)	3 (12)
None	19 (76)	22 (88)

Data are given as mean and percentages. kg: kilogram; m: metres.

**Table 2:** Comparison of lung function after VBPP and CT treatment (*n* = 15 patients per group).

FVC (L)				FEV1 (L)			FEV1/FVC (%)		
	Predicted	% Predicted	LLN	Predicted	% Predicted	LLN	Predicted	% Predicted	LLN
VBPP Mean (SD)	4.89 (0.35)	90.95 (5.34)	4.20 (0.76)	4.09 (0.41)	88.48 (5.71)	3.28 (0.87)	83.66 (5.88)	97.09 (2.22)	74 (0.76)
CT Mean (SD)	4.88 (0.33)	91.19 (4.86)	4.31 (0.77)	3.82 (0.44)	89.64 (4.12)	3.3 (0.89)	78.54 (9.01)	98.47 (4.58)	73.8 (0.74)
*p*-Value	0.9364	0.8985	0.6967	0.0931	0.5286	0.9018	0.0759	0.3026	0.4713

All data are presented as mean and standard deviation (SD). FVC: forced vital capacity; FEV1: forced expiratory volume in one second; FEV1/FVC ratio: the percentage of the FVC expired in one second; LLN: lower limit of normal (defined as below the fifth percentile of spirometry data obtained from the Third National Health and Nutrition Examination Survey); L: litre; VBPP: VATS–bullectomy with partial pleurectomy; CT: chest tube. A *p*-value < 0.05 indicates statistical significance.

**Table 3:** SF-36 results of patients who underwent VBPP compared to CT patients and the normative data from the general German population.

SF-36 Domains	VBPP Group (*n* = 25) Mean (SD)	CT Group (*n* = 25) Mean (SD)	*p*-Value	German Normative Data Mean (SD)	*p*-Value (Difference) VBPP vs. German Normative Data
Physical functioning (PF)	90.6 (9.5)	91.2(7.4)	0.8043	94.4 (10.8)	0.0841 (−3.8)
Role physical (RP)	87.0 (26.1)	88.25(24.1)	0.887	90.8 (24.6)	0.4510 (−3.8)
Bodily pain (BP)	78.8 (23.2)	81.96 (22.3)	0.6260	75.4 (22.5)	0.4604 (+3.4)
General health (GH)	69.8 (15.6)	73.28 (15.0)	0.4227	73.0 (16.8)	0.3502 (−3.2)
Vitality (VT)	54.4 (20.2)	58.24 (17.5)	0.4760	61.4 (17.0)	0.0460 * (−7.0)
Social functioning (SF)	88.5 (13.5)	87.66 (13.0)	0.8319	89.0 (16.4)	0.8807 (−0.5)
Role emotion (RE)	89.3 (18.6)	88.25 (15.6)	0.8297	92.5 (20.6)	0.4456 (−3.2)
Mental health (MH)	67.2 (14.5)	69.4(13.8)	0.5920	74.2 (15.5)	0.0271 * (−7.0)
Physical component summary score (PCS)	52.4 (6.3)	53.47 (8.5)	0.6154	52.3 (6.9)	0.9433 (+0.1)
Mental component summary score (MCS)	46.1 (7.9)	47.65 (7.2)	0.4578	50.6 (7.8)	0.0049 * (−4.5)

Data are given as mean and standard deviation (SD). A * *p* value < 0.05 displays statistical significance. SF-36: Short -Form 36; VBPP: VATS–bullectomy with partial pleurectomy; PCS includes RP, GH, PF, and BP; MCS includes SF, RE, MH, and VT.

## Data Availability

The data presented are included in this study; additional data may be provided by the corresponding author on request.
